# Association of *MGMT* promoter methylation with tumorigenesis features in patients with ovarian cancer: A systematic meta‐analysis

**DOI:** 10.1002/mgg3.349

**Published:** 2017-11-30

**Authors:** Baoli Qiao, Zhenyu Zhang, Yanfang Li

**Affiliations:** ^1^ Department of Gynaecology and Obstetrics Beijing Chao‐Yang Hospital Affiliated to Capital Medical University Beijing China

**Keywords:** clinical features, methylation, *MGMT*, ovarian cancer

## Abstract

**Background:**

The *MGMT* is a key tumor suppressor gene and aberrant promoter methylation has been reported in many cancers. However, the relationship between *MGMT* promoter methylation and ovarian cancer remains controversial. This meta‐analysis was first conducted to estimate the clinical significance of *MGMT* promoter methylation in ovarian carcinoma.

**Methods:**

Literature search was performed in the PubMed, Embase, EBSCO and Cochrane Library databases. The pooled odds ratio (OR) and their corresponding 95% confidence interval (95% CI) were summarized.

**Results:**

Final 10 studies with 910 ovarian tissue samples were included in this meta‐analysis. *MGMT* promoter methylation was significantly higher in ovarian cancer than in normal ovarian tissues (OR = 4.13, 95% CI = 2.32–7.33, *p *<* *.001). The *MGMT* had a similar methylation status in cancer versus benign lesions and low malignant potential (LMP) samples (OR = 2.01, 95% CI = 0.67–6.04, *p* = .212; OR = 1.42, 95% CI = 0.46–4.40, *p* = .543; respectively). *MGMT* promoter methylation was correlated with pathological types in which it was significantly lower in serous cancer than in nonserous cancer (OR = 0.29, 95% CI = 0.14–0.59, *p* = .001). The methylation of the *MGMT* promoter was not associated with clinical stage and tumor grade (OR = 1.46, 95% CI = 0.71–3.02, *p* = .301; OR = 1.13, 95% CI = 0.51–2.46, *p* = .767; respectively).

**Conclusions:**

*MGMT* promoter methylation may be correlated with the tumorigenesis of ovarian cancer. It was associated with tumor histotypes, but not correlated with clinical stage and tumor grade. More prospective studies with lager sample sizes are necessary in the future.

## 1 INTRODUCTION

Ovarian cancer is the second most frequently and the most deadly gynecological malignancy among women (Siegel, Miller, & Jemal, 2016). According to global statistics, approximately 238,700 new cases were clinically diagnosed with ovarian carcinoma, and it killed 151,900 cases in the world in 2008 (Torre et al., 2015). Due to difficulties of early detection, most patients with ovarian cancer are diagnosed with high stages of this disease. Five‐year survival rate at advanced‐stage ovarian carcinoma remains <20% (Heintz et al., 2006; Kaja et al., 2012). Serous histology is the most common ovarian cancer, and other histotypes include mucinous, endometrioid, clear cell tumors, and undifferentiated carcinomas, etc. (Vang, Shih Ie, & Kurman, 2009).

Epigenetic modifications, especially DNA methylation, a commonly observed epigenetic change, have been noted in the initiation and progression of human cancer (Ghavifekr Fakhr, Farshdousti Hagh, Shanehbandi, & Baradaran, 2013; Huang et al., 2015; Ma, Wang, Zhang, & Gazdar, 2013). Aberrant promoter methylation of tumor suppressor genes (TSGs) is the most common methylation in many types of cancers (Smith et al., 2016; Yokoyama et al., 2016). O6‐methylguanine‐DNAmethyl‐transferase (*MGMT*), located on 10q26, a DNA repair gene, protects cells against the effects of alkylating agent chemotherapy by eliminating the alkylation of the O6 position of guanine (Esteller et al., 2000; Tano, Shiota, Collier, Foote, & Mitra, 1990). *MGMT* is widely methylated in the promoter region in various carcinomas, including ovarian cancer (Chaudhry, Srinivasan, & Patel, 2009; Hochhauser et al., 2013; Pierini et al., 2014; Schiffgens et al., 2016). Nevertheless, data with regard to the methylation levels of the *MGMT* promoter were inconsistent in ovarian cancer. No methylation rate of the *MGMT* promoter was detected in patients with ovarian carcinoma by Agostini et al. (Agostini et al., 2016). However, An et al. reported that *MGMT* promoter methylation had a high rate in ovarian cancer (An et al., 2010).

On the basis of numerous studies with small sample size and the different methylation frequencies, we carried out this meta‐analysis to better determine the relationship between the methylation of the *MGMT* promoter and ovarian cancer in the comparison of cancer and different control groups. Additionally, we also analyzed whether *MGMT* promoter methylation was associated with clinicopathological characteristics.

## 2 MATERIALS AND METHODS

### 2.1 Literature search

The PubMed, Embase, EBSCO, and Cochrane Library databases were systemically searched to identify the studies of the eligibility using the following key words and search terms: (O‐6‐methylguanine‐DNA methyltransferase OR MGMT) AND (ovarian OR ovary) AND (cancer OR carcinoma OR tumor OR neoplasm) AND (methylation OR DNA methylation OR hypermethylation OR epigenetic silencing OR epigenetic inactivation). The search strategy was updated prior November 1, 2016. Manual search from reference lists of eligible studies was also conducted to achieve other potential articles.

### 2.2 Inclusion criteria

The following selection criteria were used for eligible studies: (i) the patients had a diagnosis with ovarian carcinoma using histopathological examination; (ii) studies must have sufficient information with regard to the methylation data to evaluate the correlation between *MGMT* promoter methylation and ovarian cancer in cancer versus control groups; (iii) studies provided data about the relationships between *MGMT* promoter methylation and clinicopathological features of patients with ovarian cancer; (iv) if authors published more than one paper using the same sample data, only paper with more information was included in the current meta‐analysis.

### 2.3 Data extraction

According to the selection criteria, we extracted necessary data from original articles: first author's surname, year of publication, country, ethnicity, type of sample, sample size, detection methodology of methylation, rate of methylation, total number of cases and controls, and clinicopathological parameters such as tumor grade, tumor stage, and pathological types. Control groups included low malignant potential (LMP) tumors, benign lesions and normal samples.

### 2.4 Statistical analysis

The pooled data were analyzed using the Stata statistical software (version 12.0; Stata Corporation, College Station, TX, USA). The correlation between *MGMT* promoter methylation and ovarian cancer in cancer versus control groups was calculated by the combined odds ratio (OR) and corresponding 95% confidence interval (95% CI).

In addition, we also assessed the association of *MGMT* promoter methylation with clinicopathological characteristics in patients with ovarian cancer. The degree of heterogeneity was detected using the Cochran's *Q* test and *I* ^2^ statistic (Coory, 2010). The random‐effects model was used when substantial heterogeneity existed (*I* ^2^ ≥ 50% and *p *<* *.1), whereas a fixed‐effects model was applied when there was no obvious evidence of heterogeneity (DerSimonian, 1996; Higgins, Thompson, Deeks, & Altman, 2003).

## 3 RESULTS

### 3.1 Baseline characteristics of studies

**Figure 1:**
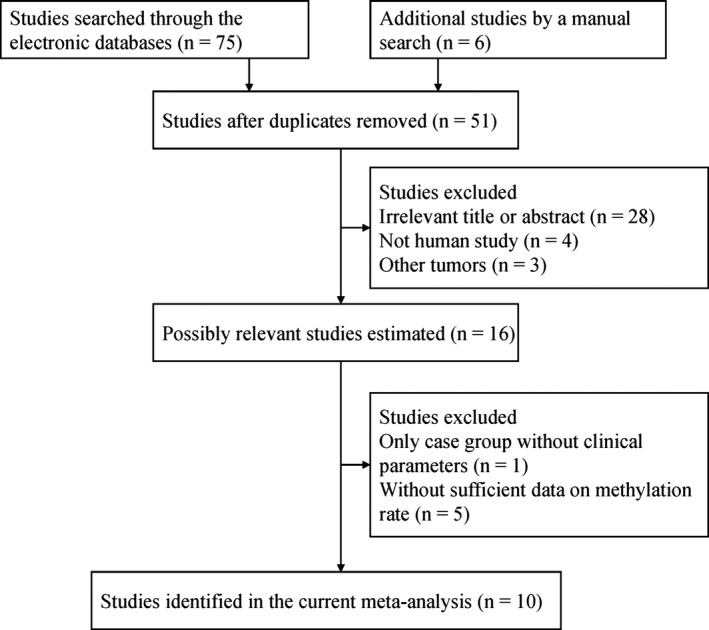
Flowchart of selection of studies

Our initial search yielded a total of 81 potential studies based on the above described search strategy. In the end, 10 case–control studies involving 910 ovarian tissue samples (Agostini et al., 2016; An et al., 2010; Brait et al., 2013; Chmelarova et al., 2012; Furlan et al., 2006; Makarla et al., 2005; Rimel, Huettner, Powell, Mutch, & Goodfellow, 2009; Roh et al., 2011; Shilpa et al., 2014; Wu et al., 2007) were included in the current meta‐analysis (Figure 1). The main characteristics of eligible studies were extracted and shown in Table 1.

**Table 1:** Baseline characteristics of the included studies in this meta‐analysis

First author	References	Country	Ethnicity	Method	Sample	Case	LMP	Benign	Normal	G3	G1–2	Stage 3–4	Stage 1–2	Serous	Nonserous
						N (M %)	N (M %)	N (M %)	N (M %)	M/N	M/N	M/N	M/N	M/N	M/N
Makarla	Makarla et al. (2005)	USA	Caucasians	MSP	Tissue	23 (8.7)	23 (0)	23 (0)	16 (0)					0/9	2/14
Furlan	Furlan et al. (2006)	Italy	Caucasians	MSP	Tissue	18 (38.9)			13 (0)						
Wu	Wu et al. (2007)	Norway	Caucasians	MSP	Tissue	52 (0)		2 (0)							
Rimel	Rimel et al. (2009)	USA	Caucasians	COBRA	Tissue	21 (4.8)			21 (0)						
An	An et al. (2010)	USA	Mix	MSP	Tissue	101 (32.7)			100 (14)			30/94	3/7		
Roh	Roh et al. (2011)	Kore	Asians	MSP	Tissue	34 (14.7)			10 (0)	0/4	5/30	3/17	2/17	0/16	5/18
Chmelařová	Chmelarova et al. (2012)	Czech Republic	Caucasians	MS‐MLPA	Tissue	69 (15.9)			40 (2.5)	6/38	5/29	9/46	2/23	5/48	6/21
Brait	Brait et al. (2013)	Brazil	Mix	QMSP	Tissue	33 (3)			13 (0)						
Shilpa	Shilpa et al. (2014)	India	Caucasians	MSP	Tissue	88 (29.5)	14 (28.6)	20 (20)	15 (0)	18/57	6/25	21/66	5/22	11/52	15/36
Agostini	Agostini et al. (2016)	Norway	Caucasians	QMSP	Tissue	126 (0)		35 (0)						0/60	0/66

### 3.2 Relationship between *MGMT* promoter methylation and ovarian cancer

**Figure 2:**
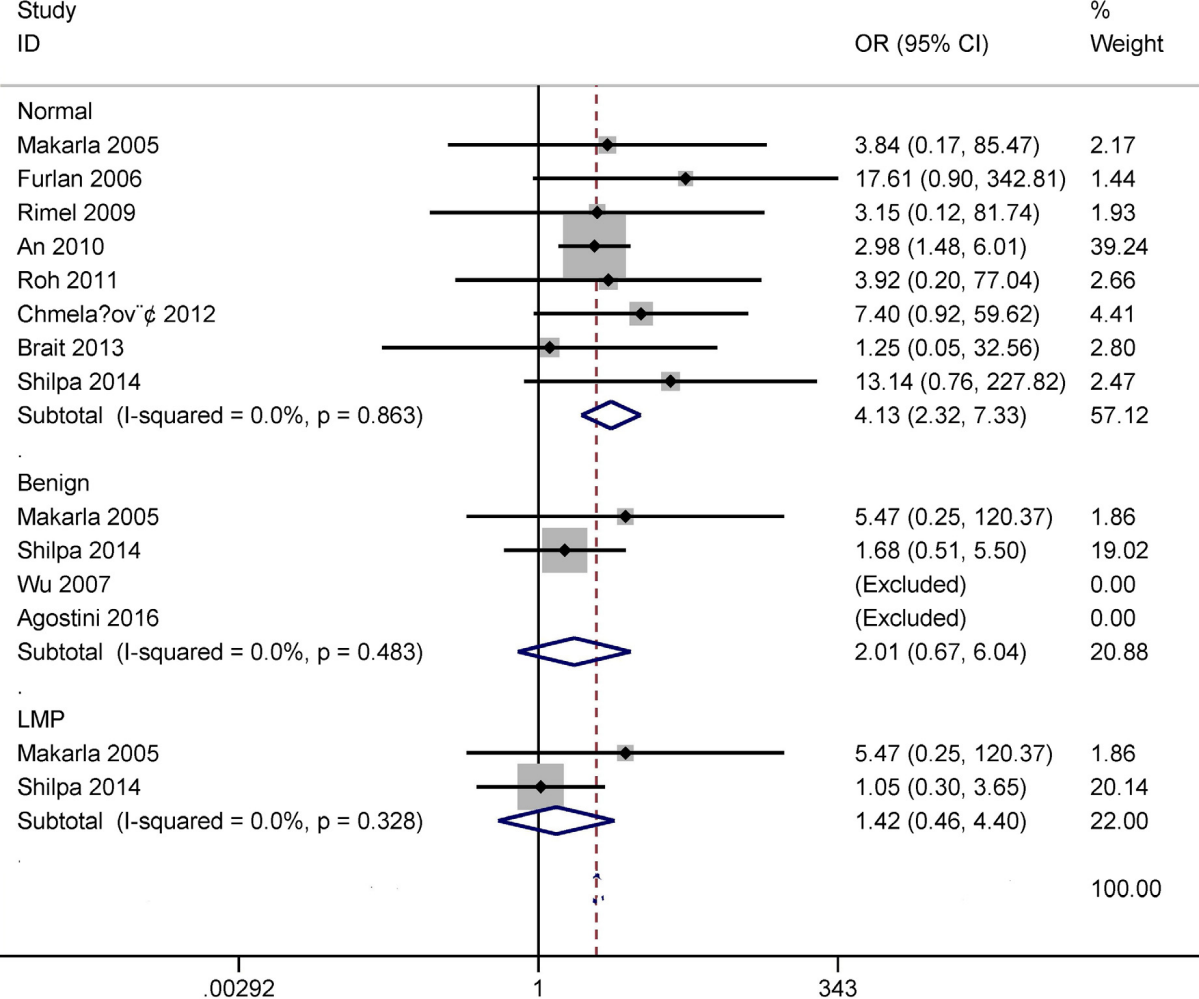
Forest plot for the association between *MGMT* promoter methylation and ovarian cancer in cancer versus LMP, benign lesions and normal ovarian tissues

Of the 10 available articles, eight studies included 387 ovarian cancer patients and 228 normal ovarian tissue samples, four studies included 289 patients with ovarian cancer and 80 benign lesions, and two studies consisted of 111 ovarian cancer patients and 37 LMP samples. No substantial heterogeneity was found in cancer versus normal ovarian tissues, benign lesions and LMP samples (all *I* ^*2*^ = 0%) (Figure 2). Thus, the fixed‐effects model was applied in this study.

The result demonstrated that *MGMT* promoter methylation was notably correlated with an increased risk of ovarian cancer in the comparison of ovarian cancer patients and normal ovarian tissues (OR = 4.13, 95% CI = 2.32–7.33, *p *<* *.001). When cancer was compared with benign lesions and LMP specimens, no significant relationship was found in cancer versus benign lesions and LMP samples (OR = 2.01, 95% CI = 0.67–6.04, *p* = .212; OR = 1.42, 95% CI = 0.46–4.40, *p* = .543).

### 3.3 Subgroup analyses of *MGMT* promoter methylation in cancer versus normal ovarian tissues

Ethnic population consisted of Caucasians, mixed population and Asians. Testing method included the methylation‐specific polymerase chain reaction (MSP) and non‐MSP.

**Figure 3:**
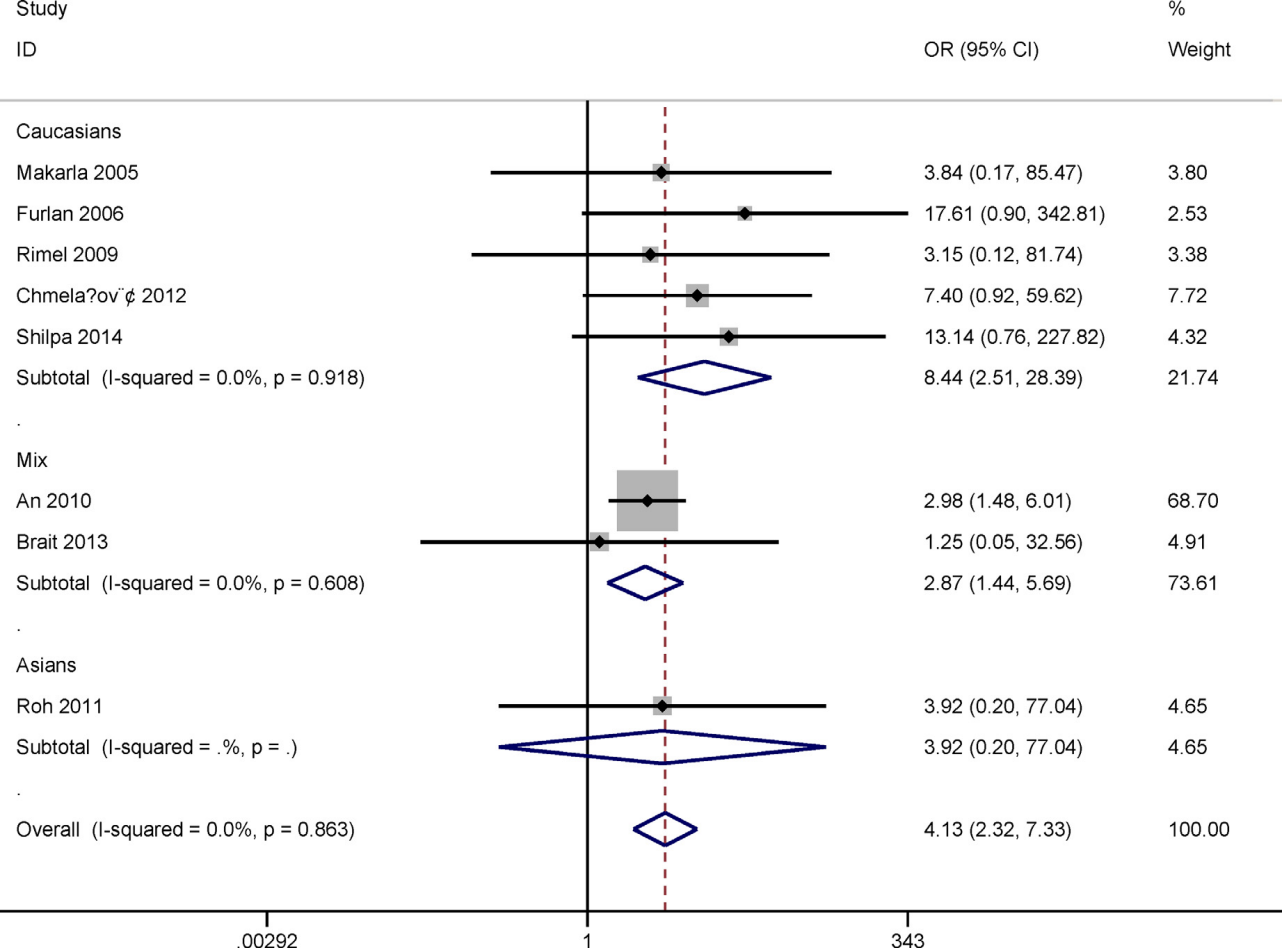
Subgroup analysis by ethnicity for the correlation between *MGMT* promoter methylation and ovarian cancer in cancer versus normal ovarian tissues

Subgroup analysis based on ethnicity demonstrated that the OR value was 8.44 (95% CI = 2.51–28.39, *p* = .001) in Caucasians among five studies, 2.87 (95% CI = 1.44–5.69, *p* = .003) in mixed population among two studies, and 3.92 (95% CI = 0.20–77.04, *p* = .369) in Asians among one study (Figure 3).

**Figure 4:**
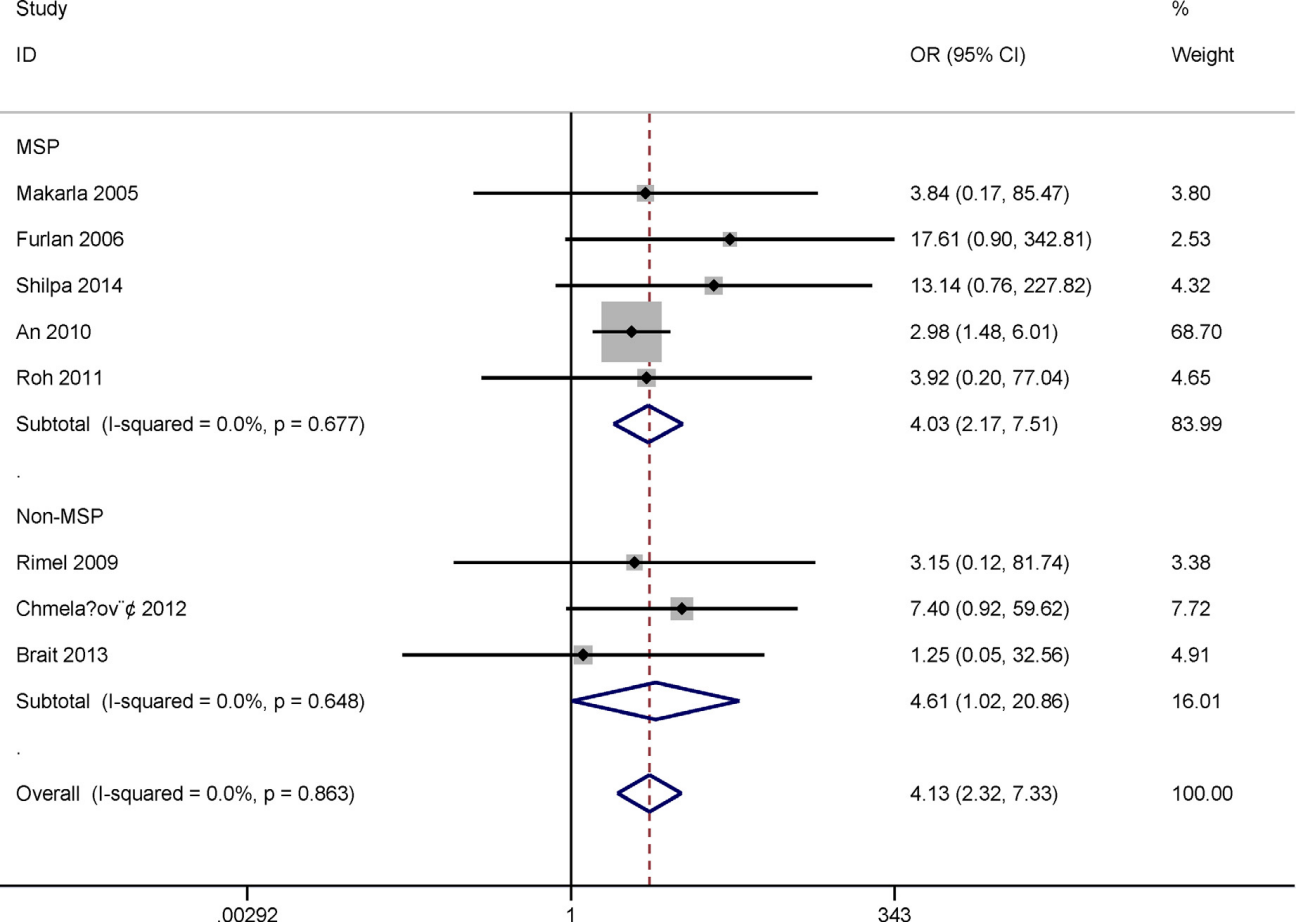
Subgroup analysis by detection method for the relationship between *MGMT* promoter methylation and ovarian cancer in cancer versus normal ovarian tissues

Subgroup analysis by detection method of methylation indicated that the OR of the MSP subgroup was 4.03 (95% CI = 2.17–7.51, *p *<* *.001) among five studies, and 4.61 (95% CI = 1.02–20.86, *p* = .047) in the non‐MSP subgroup among three studies (Figure 4).

### 3.4 Relationship between *MGMT* promoter methylation and clinicopathological features

**Figure 5:**
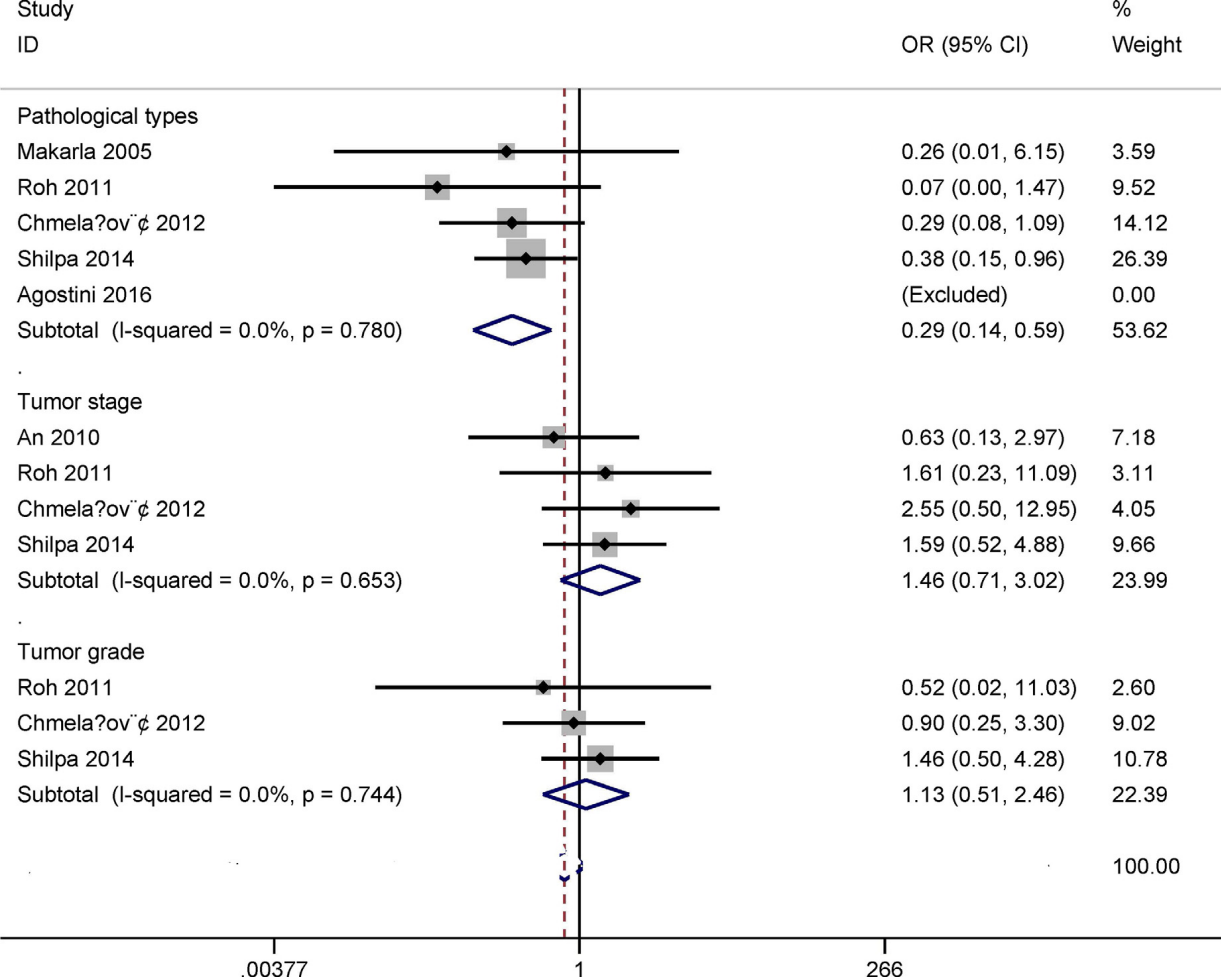
Forest plot for the association between *MGMT* promoter methylation and clinicopathological features

No evidence of heterogeneity was detected in relation to pathological types, tumor stage, and grade under the fixed‐effects model (all *I* ^*2*^ = 0%) (Figure 5).

The pooled OR indicated that *MGMT* promoter methylation was significantly linked to pathological types in ovarian cancer (OR = 0.29, 95% CI = 0.14–0.59, *p* = .001), including five studies with 185 serous cancer and 155 nonserous cancer patients.

The pooled OR showed that the methylation of the *MGMT* promoter was not correlated with clinical stage (OR = 1.46, 95% CI = 0.71–3.02, *p* = .301), including 223 patients with stage 3–4 and 69 patients with stage 1–2 ovarian carcinoma among four studies.

The pooled OR from three studies involving 99 patients with grade 3 and 84 patients with grade 1–2 ovarian carcinoma indicated that *MGMT* promoter methylation was not associated with tumor grade (OR = 1.13, 95% CI = 0.51–2.46, *p* = .767).

## 4 DISCUSSION

The methylation of DNA of promoter region is a common mechanism for the inactivation of tumor suppressor genes in cancer, which leads to the loss or reduction in gene expression (Herman & Baylin, 2000, 2003). The downregulation of *MGMT* expression through promoter methylation has been shown in various human cancers, including lung cancer, glioblastoma, gastric carcinoma, and salivary gland carcinoma (Cabrini, Fabbri, Lo Nigro, Dechecchi, & Gambari, 2015; Lee et al., 2008; Pulling et al., 2003; Yousuf et al., 2014). The association between promoter methylation of the *MGMT* gene and its expression was found in ovarian cancer, with negative *MGMT* expression (Roh et al., 2011). The methylation frequency of *MGMT* promoter was found to be different in this study. The methylation rate of *MGMT* promoter ranged from 0% (Agostini et al., 2016) to 38.9% (Furlan et al., 2006) in ovarian carcinoma. *MGMT* promoter methylation had a different methylation frequency in LMP samples, with a variation in 0% (Makarla et al., 2005) to 28.6% (Shilpa et al., 2014). The rate of *MGMT* promoter methylation was different in benign ovarian tissue samples (Agostini et al., 2016; Shilpa et al., 2014). Moreover, An et al. reported that *MGMT* promoter had a methylation frequency of 14% in normal ovarian tissue specimens (An et al., 2010). Some studies reported that *MGMT* promoter was unmethylated in normal ovarian tissue specimens (Furlan et al., 2006; Makarla et al., 2005; Rimel et al., 2009). Thus, we performed this study using eligible publications to further identify the relationship between the methylation of *MGMT* promoter and ovarian carcinoma.

No evidence of heterogeneity existed in ovarian cancer versus LMP, benign lesions and normal ovarian tissues (all *I* ^*2*^ = 0%), suggesting the stability of our results. The result showed that ovarian cancer had a significantly higher methylation status of *MGMT* promoter than normal ovarian tissues, which suggested that *MGMT* promoter methylation may be an early event of the carcinogenesis of ovarian cancer. *MGMT* promoter methylation had s similar frequency in ovarian carcinoma versus LMP and benign lesions. However, the result should be cautious based on fewer studies with small subjects when cancer was compared to LMP and benign lesions.

When cancer was compared to normal ovarian tissue samples, subgroup analyses were conducted to find the correlation in the different subgroups. Subgroup analysis of detection method demonstrated that *MGMT* promoter methylation was correlated with ovarian cancer in the MSP and non‐MSP subgroups. Subgroup analysis of ethnic population showed that the methylation status of *MGMT* promoter was associated with ovarian carcinoma in the Caucasian and mixed populations, but not in Asian population, suggesting that the *MGMT* may be a susceptible gene for Caucasians and mixed population. While the result regard the subgroup analyses should be carefully considered as only small sample size were included, particularly in mixed population and Asians subgroups.

We also determined whether *MGMT* promoter methylation had a correlation in relation to clinicopathological characteristics, including pathological types, clinical stage, and tumor grade. Our findings indicated that *MGMT* promoter methylation was significantly correlated with pathological types, and it was significantly lower in serous carcinoma than in nonserous carcinoma (OR = 0.29, *p* = .001), which suggested that *MGMT* promoter methylation had a decreased the risk of serous ovarian cancer. No significant association was found in relation to clinical stage and tumor grade.

Several potential limitations should be stated in this study. First, the search strategy was limited to available papers published in English language. Other publications published in other languages were eliminated because of the difficulties in reading. Second, based on small sample size, the results with regard to subgroup analyses should be further done in the future. Third, when cancer was compared to LMP and benign lesions, multiple studies with larger sample size should be performed to clarify our results.

In summary, our findings suggested that *MGMT* promoter methylation may play a crucial role in the initiation of ovarian cancer. *MGMT* promoter methylation was linked to pathological types, but not associated with clinical stage and tumor grade. In the future, additional prospective studies remain needed.

## CONFLICT OF INTEREST

None declared.
